# Medico-Chirurgical Transactions

**Published:** 1829-10-01

**Authors:** 


### MEDICO-CHIRURGICAL TRANSACTIONS, VOL. XV. PART. 1.

OBSERVATIONS ON LOCAL DISEASES, TERMED MALIGNANT. By *Benjamin Travers*, F. R. S. &c. &c. Part. I.

No one who observes with attentive eye the signs of the present times, professionally speaking, can fail to remark the increasing taste for morbid anatomy and pathology in this country. Time was, 'tis *not* sixty years since, when the growth and use of the articles in question were almost monopolised by France, and the English practitioner was lamentably deficient in the knowledge of disease afforded by the scalpel and the saw. We will not deny, indeed we cannot, that a portion of the old leaven still sticks to many, and the bigotted routinist is yet to be found, who professes to know little and care less about morbid anatomy and post-mortem examinations. With him the *interest* of a case is, in the Hudibrastic acceptation of the term, "just what it will bring," and when the breath is out, the patient is consigned for good and all to the undertaker and the earth-worm! We fear, however, that this sacred band is thinning daily, not so much, we allow, by desertion, as the ruthless scythe of Father Time, lopping sage after sage from its ranks. The great majority of the profession, are however, of a temper somewhat different from the select few that oppose all reform in science as innovation, all innovation as mischievous error. But we possess amongst us those who are sinning on the other side, who advance too far instead of too little, and would seem to regard the stores of information gradually formed by the labours and the labourers of twenty centuries, as so much rubbish, to be *shot* as soon as possible, in order to make room for the showy structures of modern days. These rash men, these Caliph Omars in their way, who would burn the learning of antiquity because it is not in their Koran, are ten times more active, and therefore more dangerous than the drones.

We have no wish to figure as enthusiasts, nor to rate the discoveries of the moderns for more than they are worth, but still we have no hesitation in expressing our belief that the science of medicine is improving, in spite of both the parties to whom we have alluded. Neither can it be denied, in our opinion, that the main source, the *fons et origo*, of that improvement is to be found in the cultivation of morbid anatomy, in other words the structural alterations that constitute disease. Much has already been obtained, and much is yet to be got, by diligently following this track. It is on this account that we have always endeavoured to place before our readers the researches of pathologists more fully, perhaps, than any contemporary journal of medicine, and we trust that we have not been altogether unprofitable labourers. At the same time we have been cautious to point out the dangers and delusions that must necessarily ensue from trusting too implicitly even to morbid anatomy. There are things in heaven and earth that pass our philosophy most woefully, and the phenomena of disease are assuredly amongst them. He who imagines that post-mortem researches will make all clear at the bed-side, will find himself egregiously deceived, despite of the confident and somewhat arrogant declamation of certain writers of the present day.

We were led to the foregoing remarks by the subject of the paper to which we shall direct the attention of our readers. Observations "On the Local Diseases Termed Malignant," is a title which promises but little in a curative or practical, but much in a pathological point of view. Mr. Travers is already so favourably known to the profession, as an author and a surgeon, that nothing from us could raise his reputation in either character. Whether the present paper will prove a worthy descendant of those that have previously sprung from the same pen, the public at large must decide. The *fiat* is with them, and we now proceed to place before our brethren, very many of whom will never peruse the original memoir, the materials whereon to form their judgment.

Mr. Travers, in the genuine Horatian style, rushes at once *in medias res*, and begins by informing us that chronic local diseases may be divided into tractable and intractable. Mr. Travers allows that the division is too general—we think it is fundamentally bad. A chronic disease may be tractable at one time, intractable at another; or it may be intractable merely from situation; or peculiar circumstances, general or local—in short, the exceptions to such a division are so great as to eat it to the very kernel. Why not, we would ask, be contented with the old-fashioned terms of malignant and non-malignant? We all conceive that we understand them, and that is a point of some importance. Mr. T. observes that a disease may be intractable in its *nature;* first, from its depending on a poison absorbed, as the venereal; or on a bad state of system, as the scrofulous; or a cachexy made up of both. Secondly, "from its being a disease of a part which, when it has reached a certain stage, generates a poison and thus diffuses its species and destroys contiguous textures, so that after this stage is reached, however complete its apparent extirpation or destruction, it is liable to reappear." Thirdly, from its being a disease of the constitution shewing itself primarily and exclusively in tumours, bearing a similar character, in various parts of the body, and proving within a short period destructive to life, in despite of the earliest interference.

Such are our author's definitions of diseases intractable in their nature, and if ever proof was wanting of the sandy foundation of nosology, these very definitions would afford it. The two latter classes are stated to embrace cancer, and they evidently do so. The first comprehends syphilis, scrofula, and their combinations; yet why these two diseases should enjoy such favour we cannot very clearly see. Surely the sore, or the node, or the eruption of syphilis is not *necessarily* intractable in its nature, and if *accidentally*, the whole passage is palpably absurd, for a property cannot at the same time be accidental and inherent in the nature of a thing. We hate cavilling, but would recommend Mr. Travers to re-write the commencement of his paper.* To do him justice, he appears to feel the pressure of his incubus, for he quickly drops the term intractable (a bad one in every respect) and proceeds to the consideration of "the diseases termed malignant," i. e. incurable, and leading sooner or later to the patient's destruction.

---

* The fact is that it had better be omitted altogether, for assuredly it explains nothing, leads to nothing, has no natural connection either with the title or the body of the paper, and is only a clumsy kind of prologue after all.

It becomes a serious question whether a disease be malignant on its first appearance, or grows so in the course of time, having arisen under circumstances favourable to the attainment of malignity. If the former be the fact, it is, è principio, a disease of the constitution, whence alone the character of malignity can be derived. The following views are just, but they must be *studied* to be perfectly understood; *reading* them is not enough.

"It is conceivable that a simple local disease may become malignant by the influence of the constitution upon it, as a simple fever may become typhoid or putrid, but a strictly local affection cannot be malignant. When we speak of the decided malignity of a tumour, or ulcer, we mean to say, that it is such a disease of the system shewing itself in a part. When we say that it has a malignant aspect, or resembles a malignant tumour or ulcer, we mean that it is analogous to those in which the constitution, sooner or later, takes such an action. We cannot well conceive of malignity, as the exclusive or innate property of a part. A change of structure, whether of increase or loss of substance, which not only resists every remedy, but which, being extirpated or destroyed, is reproduced either in the vicinity or at a distance from the original site, is certainly not, in strictness, a local disease. But if from any local cause a sore refuses to heal, or falls into gangrene; if by the extension of the ulcerative process blood-vessels are opened and fatal hæmorrhage ensues; if by the profuseness of a secretion the patient dies exhausted; if by the incessant irritation of the nervous system, or the morbid actions set up in vital organs under a protracted symptomatic fever, life is extinguished; the disease does in no respect imply a malignant nature, though often so considered, malignant diseases being subject to a similar termination. It is to incurableness from causes not local, and consequently the disposition to appear in more than one part at the same time, or to reappear when the first affected part has been freely removed, that the term malignity is applicable.

"If a local disease were from its earliest germ impressed with a malignant character derived from a morbific matter in the constitution, it would be so much a constitutional disease, that the removal of it could never be urged on the ground of permanent benefit. To one species of malignant disease this character is applicable, as I shall explain hereafter.

"But although a local disease, strictly speaking, cannot be malignant, it is clear that a disease, altogether local in its commencement, may in its progress stir up an action of the constitution which imparts to it that character, or a malignant constitutional disease may only shew itself in a part.

"Again, constitutional malignancy may not shew itself in any part by a specific organic change, as is the case with some poisons and contagious fevers; whereas the disease of a part must derive its malignant property from the constitution." 199.

The inference drawn from the preceding observations, is this; that the scirrhous tumour may be, "and undoubtedly is," in the first instance, a local and single disease. The proof of the assertion will be found, according to our author, in the record of "a thousand instances of the early and complete removal of the disease, without threat of return during many years of the patient's after life." This may even be the case under otherwise favourable circumstances in the ulcerated stage. We laud Mr. Travers's premises, but dissent in a great degree from his conclusions respecting the non-malignity of the scirrhous tumour. It is a flattering tale that we are told, but sad experience compels us to attach light faith to the thousand successful operations for cancer upon record! If Mr. Travers can but prove the soundness of his views respecting cancer, he will assuredly confer a boon upon science and humanity, that must immortalize his name. But we dare not hope it. Mr. Travers writes that "to *incurableness from causes not local*, and consequently *the disposition to appear in more than one part at the same time*, or to *reappear when the first affected part has been freely removed*, that the term *malignity is applicable*." Surely the scirrhous tumour answers to these characters. However, our author considers the question more fully farther on, and we drop the discussion for the present. We shall meet again at Philippi!

"We may consider carcinoma as a genus of the order 'malignant diseases.' Its species are 1st the scirrhous, 2d the medullary. Their respective modifications and varieties are to be referred to those of structure. The first has been also called the 'hard' or the 'stone' cancer—the second, the 'soft' cancer, 'fungus hæmatodes'—'encephalodes'—'melanodes'—'fungoid inflammation'—'medullary sarcoma,' &c. &c. according to its varying phenomena of colour and consistence. The inconvenience of a change, or a multiplicity of name, is far greater than the retention of one that might admit of improvement. Medullary is the most descriptive and comprehensive definition; I shall therefore retain it." 201.

Whether Mr. Travers does well in considering scirrhus and medullary sarcoma, or fungus hæmatodes, as species of the same genus, carcinoma, we shall not take upon us to determine. Certainly all the forms of malignant degeneration evince a very marked disposition to run into one another, and scirrhus and fungus hæmatodes have been found to exist at the same time in different parts of the same individual. Notwithstanding this, however, we had rather not lump them together under the indefinite term of cancer, because they materially differ in structure, progress, and even malignity. In our own opinion, nosological arrangements of local diseases throw open a door for vagueness both of observation and description. If a man considers many affections as merely varieties of one, he is naturally apt to attach less importance to their discrimination, by which means pathology, and ultimately perhaps even practice, is the sufferer. Our author proceeds to treat at length of the *scirrhous cancer*, or in common parlance, scirrhus.

It originates in some or other description of secretory structure, as 1. the follicles of the mucous membrane; mouth, fauces, stomach, and intestinal canal: 2. of the reflected integument at the orifices of the uterus, vagina, anus, urethra: 3. of the skin, especially where they most abound; as the face, scrotum, prepuce: 4. the secreting glands termed conglomerate, as the lachrymal, salivary, mammary,—the liver, pancreas, testicle: 5. the lymphatic, absorbent or conglobate glands. Mr. T. is not aware that it originates in other textures, and proceeds to consider the cause of the exclusive liability of secretory structures to scirrhus. Passing over the theories with which he contends, and which, it must be owned, are unsatisfactory enough, we must mention the one advanced by himself.

"Glandular organs are the seat of scirrhus, because they are more abundantly than other parts supplied with vessels whose office is the separation and combination of new materials from the circulating fluid; and it is sufficiently probable, that when, having been habitually and actively employed, they cease to be so, these vessels make preternatural deposits, and expend their energies, wantonly as it were, upon new and useless structures. This seems to me to be the origin of the scirrhous tubercle. The dilatable tissues—those most subject to changes of capacity under great diversities in the quantity and impulse of the circulation, and necessarily furnished with a very abundant cellular tissue, common or proper—are peculiarly subject to originate scirrhus.

"But it must at the same time be admitted, that the disease often arises in those individuals, in whom the organs most frequently visited by the complaint have not been subjected to extreme variations of capacity, as in the virgin female." 204.

We fear that Mr. Travers' bullet has scarcely hit the mark much closer than its predecessors. The cessation of secretion is a condition of things which seldom obtains in any cases of scirrhus but that of the female, at the termination of the catamenia. How is cancer of the lip, &c. in men, to all appearance healthy, explained by this theory? Mr. Travers having dwelt upon the *seat* of scirrhus, next considers the *time* of its occurrence. He remarks its rarity before the forty-fifth year, its prevalence in females at "the turn of life," and seems to hint obscurely at a coincidence between it and the osseo-cartilaginous deposits in the coats of the blood-vessels, deposits which, with other changes in the body, may be viewed as climacteric, and evince the decline of the circulating power. Such changes he imagines to be connected with a sluggish and feeble, perhaps obstructed state of the capillary, vascular, and absorbent circulation.

"*Structure of Scirrhus.—External characters*—hardness with increase of weight, inelasticity or toughness in some cases, knotty or craggy induration in others. Circumscription, and mobility beneath the skin in its earliest stage, but not to such a degree in the subjacent bed as to allow of the fingers passing beneath the tumour, and turning it edge upwards. Next, i. e. in the second stage, close adhesion to the tegument, and such incorporation with the glandular organ in which it is seated, as to have no mobility but that of the gland itself upon the parts beneath. The adhesion of the skin, either stretches, or partially retracts and puckers it, according to the smooth or unequal surface of the tumour, and to the close or loose attachment, and particular conformation of the integument at the spot, as for example, next the nipple and at a distance from it; or beneath the mucous membrane of the pylorus or rectum, and the common integument of the body. Third stage: Contraction and diminution, by pressure, of volume in the gland as the tumour increases. Abrupt projection of one large coloured tubercle, sometimes of several smaller tubercles or nodules. Irreducibleness of volume and hardness by topics or medicine. Transient pains, which have been hitherto obscure and occasional, now more distinct and frequent, like the pricking of a sharp instrument, with a sense of heat or burning. Dusky or livid red colour of the skin, with resplendent tension. Excoriation or cracking of the skin at the summit or base of the tubercles, and fungous elevations, with ichorous and sanious oozing." 207.

Mr. Travers justly observes that no one or two of the foregoing symptoms are diagnostic of scirrhus, but that the assemblage of the whole must be admitted to describe it and nothing else, although they constitute only the external characters. There is a tumour met with in the breasts of young women, resembling a shelled walnut in size and nodosity, of stony hardness, and irreducible by pressure, mercury, iodine, or blisters. It depends on enlargement and partial adhesion of the lactiferous tubes in a cluster, and must not be mistaken for scirrhus. Mr. T. has also met with a deep-seated, partially elastic swelling, with an uniform smooth surface, in consistence like cartilage, not painful, but gradually increasing and involving by adhesion the skin and the neighbouring parts. In this state it is a solid, unyielding, immoveable mass, which shews no disposition to suppurate, and is quite irreducible. It occurs in all ages, and often destroys life by compression of the first passages, as the larynx, œsophagus, &c. Upon section, it resembles the ovarian tumour, or the "potatoe-tumour" of the uterus; the osteo-sarcomatous tumour of the ribs, ossa innominata, and other bones. It has a dense and uniform interior. Mr. Travers does not mention in what structure this tumour has its origin.

"*Internal Characters of the Scirrhous Cancer.* These, though presenting some variety, are more to be depended upon (than the external.) In the first stage, on section, a tough, inorganizable, and pretty compact mass, of a white and yellow-brown colour, smooth and moistened by a slightly unctuous fluid; its consistence is not uniform, being hard in the centre, so as to form a nucleus. The circumference is defined by the termination of red vessels forming a vascular boundary. Upon floating in water, and still more by a certain maceration, the texture opens so as to bring into view concentric areolæ, having their interstices filled by a white granular matter, which may be picked out from the meshes. These areolæ are crossed by faint white lines, at irregular intervals, in the direction of radii from a centre, visible to the naked eye, and very conspicuous under a magnifier, giving the section some analogy to that of a lemon.

"In the second stage, when inflammatory action commences, and is announced by shoots of pain, the relative firmness of the centre and circumference of the tubercle becomes reversed, the centre being pulpy or broken, while the circumference retains its firmness. The surrounding parts are now found to have lost their natural elasticity by condensation of texture, and partake of the firmness and weight of the scirrhus, giving considerable apparent increase of volume to the tumour, which is now less defined at its margin, and, in fact of a compound character. The dense opaque white lines which, traversing the tumour in the direction of radii, diminish in density as they proceed outward, and are lost in the extreme circumference of the gland, are not the production of disease, but the septa, which divide and support the lobules of which the gland is composed, in an opaque and thickened state. Within the wall of the tubercle, one or more cysts, containing a dark yellow or coffee-brown fluid, are sometimes met with, but are often not present.

"The above appearances are characteristic of the genuine scirrhus. The softened and broken down nucleus bears little analogy to the appearance of imperfect and caseous suppuration in the centre of diseased mesenteric glands, or other scrofulous swellings. The peculiar firmness of the exterior; the entire absence of inflammatory effusions, membraneous lymph, and purulent matter; and the involvement of surrounding parts in the same compact and indurated state,—cellular membrane, skin, and even muscle,—are sufficient to distinguish it. The lymphatic glands in the vicinity are enlarged, but present a very inferior degree of hardness, and on section are found to have retained their natural colour and structure for a considerable time. The septa are conspicuous, as before described, and eventually, the centre and surrounding parts undergo the same change as was observed in the texture originally affected. By extension, no parts are excepted from contamination in the progress of a scirrhus; but neither the mammary, the lymphatic, nor other glands have any original substantive share in the disease,—it is a new and interstitial deposit." 210.

Our author brings forward the scirrhous tubercle of the liver, exemplifying not merely the insulated formation of the disease, but its stages and progress. But to proceed with the thread of the description, the ulcerative process at length opens the tumour where the cracked and livid integument, previously exuding a sanious ichor, is most prominent, and partial sloughing of the integument sometimes assists in enlarging the external opening.

"Suppuration now takes place in the surrounding cellular membrane, and as granulations spring up luxuriantly from the sides, the centre of the tumour gapes and becomes a cavity more or less considerable. The granulations have a spongy or fungoid character, and are so elevated and broadly everted as to give the appearance of additional depth and breadth to the sore. As the sloughing process enlarges and deepens the centre, the disease becomes exceedingly offensive, and although granulations continue sprouting circumferentially at the same time, they have not the power of maintaining their vitality. It is in vain that we attempt to preserve the ulcer from foulness and fetor by detergent applications; we can render it clean, but in a day or two the newly cleansed surface ulcerates afresh, instead of advancing towards cicatrization." 212.

The wound of the operation (supposing such performed) heals kindly, but after a time an indurated tumour steals into notice at the angle of the cicatrix, or near it, and again marches on like the original growth. Mr. T. observes that in no one stage is there any analogy to scrofulous action, a sentiment to which we must all respond. A chronic abscess of the breast has been frequently mistaken for scirrhus, and not long ago we saw a breast removed in consequence. It occasionally forms between the gland and the pectoral muscle, "when it lifts the gland, and may be felt obscurely at its edge."

"In scirrhus it is not suppuration which, as in scrofulous and other tumours, brings the disease to the surface, nor does the external skin ulcerate, until after the scirrhus. Its first and proper action is within itself. Encompassed by a dense wall, the centre breaks up by ulceration, constituting the state of occult cancer. Indeed the scirrhus is seldom removed so early as to be found with its nucleus unbroken. A sudden and extensive gangrene has been known to eliminate the disease completely, and upon this principle the cautery, arsenic, and other corrosives, are sometimes successfully employed by cancer curers. Mr. Cline had a patient in St. Thomas's Hospital many years ago, in whom the sloughing process went on spontaneously under a linseed meal poultice, to such extent, that the wound afterwards healed soundly. I have seen more than one case in which extensive cicatrices of ulcers existed with much puckering and stretching of the skin of the chest, and no vestige of the breast remained. In one of these the patient, a lady residing in Berkshire, resisted the pressing advice of a consultation of London surgeons to allow of the extirpation of the tumour many years since. She has been in the constant habit of taking the medicine then prescribed, the extract of hemlock, almost *ad libitum*, with which she supplies herself regularly from Apothecaries' Hall. She is still a stout, healthy-looking person, as formerly, and attributes her cure to the medicine." 214.

Mr. Travers alludes to the occasionally dormant condition of the scirrhous tumour, even to the close of the patient's life, and cites a case of his own in illustration. The occasional, though certainly too rare, removal of the scirrhus by sloughing, and its remaining through a period of many years inoffensive to the system, are considered by our author as "additionally conclusive as to the original strictly local character of the disease." Sometimes the ulcerative action, instead of excavating the breast, creeps upon the surface, and converts the entire front or side of the chest into a warty or tubercular ulcer, discharging profusely thin and acrid matter, whilst the subjacent tumour is large, hard, and immoveably fixed. In these cases the sufferings are great, the skin is "charged to saturation with the poison, a pimple, mole, or leech-bite being often converted into a tubercle, and not unfrequently the opposite breast becomes affected, and an inflamed absorbent or a chain of small tubercles extends across the sternum from one to the other." In other cases there are oblong folds and furrows of the skin, looking extremely like cicatrices, and depending, in our author's opinion, "on an interstitial, instead of ulcerative absorption, and partial but firm lines of adhesion between the skin and the pectoral muscle." The permanent obstruction or obliteration of the absorbents, together with the disease of the lymphatic glands, gives occasion to that enormous œdematous swelling of the inferior parts, which is among the last symptoms of this dreadful disease.

### MEDULLARY CANCER.

The differences between this and "Scirrhous Cancer," are so briefly and well pointed out by Mr. Travers, that we cannot do better than transcribe them.

"This species, also a morbid growth, differs from the scirrhous cancer, 1st. In its property of affecting any or all textures without exception, skin, bone, muscle, fascia, tendon, ligament, nerve, membrane and gland of each description, blood-vessel, and all the viscera and organs of sense.

"2d. In travelling as much by the blood-vessels as the absorbents of the part, and therefore not affecting contiguous parts only, but remote districts of the body; structures altogether dissimilar, and often, many at the same time.

"3d. In external characters—which, however modified by structure, situation, and connection of parts, are the reverse of those of scirrhus, the tumour having a degree of elastic softness which conveys a sensation of fluidity, a uniform roundness, and a volume far exceeding that of scirrhus, and, in short, more resembling a deep collection of matter.

"4th. In internal characters—being uniformly lardaceous on section, or pulpy, or semi-fluid, and intimately blended with original textures, i. e. deposited in their interstices and proper cellular tissue. By its seat it is modified in consistence, being in bone of so much firmness as to be osteo-sarcomatous in appearance; also in colour, as in the eye and some other parts, hence the term 'melanodes;' and in the limbs where it reaches a formidable magnitude, being mixed up with the coagula of blood which has escaped from the veins destroyed by it, 'hematodes.' The extreme states of a fresh and a decomposed brain present its different degrees of consistence; and its colour upon section is either opaque white or black, or a mixture of the two, a mottled grey and brown, or cineritious.

"5th. It differs last, but not least, from the scirrhous species, in being the disease of early rather than advanced life, and from the first a truly malignant and therefore a constitutional disease." 217.

It cannot fail to be remarked, that many theoretical notions are mixed up with the enumeration of purely structural distinctions in the foregoing passage. Take, for instance, the second head, where the medullary cancer is said to differ from the scirrhous, in "travelling as much by the blood-vessels as the absorbents of the part, and *therefore* not affecting contiguous parts only, but remote districts of the body, &c." It is obvious that this is mere speculation, and neither supported by, nor capable of, proof. The explanation, too, of the occasional *melanoid* colour and *hæmatoid* consistence, by the *site* of the tumour, will not bear investigation.

Like scirrhus, the medullary cancer is unconnected with a suppurative process, but its characteristic action is first adhesive, then ulcerative, and seldom associated with gangrene. At the same time, we would observe that the medullary tumour occasionally sloughs, and that to a considerable extent, a process which, though not identical with mortification, is yet allied to it. Mr. Travers remarks, that he has never known a patient survive this disease, after its removal by the knife, for a longer period than four years, and instances several cases illustrative of the observation. We can bear our testimony to the comparatively rapid return of this disease after the operation, and no doubt the voice of the profession will echo our author's melancholy prognosis.

The scirrhous cancer also develops itself at times, in remote organs, after the removal, and without the return of the external tumour, but by no means so often as the medullary disease. When a scirrhous tumour has passed into ulceration, the operation is seldom successful.

"There is, however, a great and obvious difference in the sympathy of the constitution with scirrhus in different individuals. I should say there were two classes of cases, one in which the constitution might either be supposed to labour with the *materies morbi* before its local manifestation, or which, by the general and simultaneous failing of the functions of health, appears to invite or to be incapable of resisting the assault. As when mental disquietude, bodily fatigue, impaired digestive power, wasting and muscular flaccidity, sallow and haggard countenance, prurient affections, or erythematous inflammations of the skin precede the accidental discovery of a small lump in the breast. In precisely such a case I was consulted, within six weeks from the discovery, by a widow lady of 57 years, who had suffered from a domestic affliction severely a year before, and upon immediate conference with Mr. Cline, the opinion given was decidedly adverse to the operation, although the local circumstances were yet as favourable as could be conceived. She died within two years, the disease running through all its stages.

"In the other and more frequent case, the actual enjoyment of health appears not to be incompatible with the slow progress of the scirrhus even for years, of which the pain or rather uneasiness is so inconsiderable as scarcely to draw attention to it. Somewhat suddenly it becomes more uneasy from increase of size and weight, a short acute darting pain with a flush of heat is experienced at lessening intervals, and although the health is not very noticeably disturbed before the skin inflames and threatens, or begins to ulcerate, yet, in despite of strong animal spirits, there are obscurer indications to the medical observer—in the sallow complexion and lack-lustre eye, the languor and frequent head-ache, defective appetite and rest, with transient heats and wandering pains in different parts of the body, and especially in the loss of volume and firmness in the muscular flesh—that the poison is at work and circulating with every pulse.

"Where the constitution is affected as in the case first described, it is at least predisposed to be sooner irritated by any local malady, and to favour, rather than otherwise, those changes by which the deleterious qualities of morbid secretions are evolved, and the more rapid career of the disease to its termination is thus explained; where, on the other hand, the constitution is healthy and even vigorous at the time of the formation, its power of resistance both to the local irritation and to the changes which precede and promote the admission and action of the poison, is in proportion great, and the progress of the disease tardy." 221.

Mr. Travers is of opinion, that "scirrhus becomes a constitutional disease" long before any external ulceration takes place, and instances the early affection of the contiguous absorbent glands, as proof of the fact. It is certain that this glandular contamination materially lessens the chance of success from an operation; but it is equally certain, that comparatively few cases are the subject of consultation in which it is not more or less apparent. Mr. T. does not deem it, *per se*, a bar to the operation, for "the degree of induration and the state, especially the freedom from adhesions, of the principal tumour," must likewise be thrown into the scale. Mr. Travers does not deny that success has sometimes followed the operation when the cancer was already open, but still very judiciously throws these rare exceptions overboard, in deciding on the vital principle in the treatment of true scirrhus—its earliest possible removal.

### Actual Origin and Nature of Cancer.

Mr. Travers observes, and probably with justice, that the *formation* and circulation of a poison in the blood, is the only rational mode of explaining the simultaneous or successive appearance of the same malignant disease in remote parts of the same individual. If this be granted, it follows, of course, that exclusively *local* contamination argues the non-constitutional origin; and diffused contamination the constitutional origin, of the disease. All this is fair enough, and consistent with sound reasoning; but the practical question resolves itself from a logical or metaphysical subtlety into plain matter of fact—is scirrhus in one or in the other of these classes; is it a constitutional disease or is it not? Mr. Travers, we imagine, sees the great difficulty of answering the question decidedly, but the following passage contains the pith of his opinion. The replies, we must confess, are somewhat in the ambiguous tone of the Pythia of old, but still they evidently shadow forth our author's idea upon the subject.

"Nor is it consistent with ordinary observation that the poison acts upon the system during the integrity of the tubercle, since persons generally recover, and finally, in whom the disease is freely removed in this early stage. On the contrary, if the tubercle be softened and undergoing ulceration, *i. e.* absorption, the disease recurs, however freely the part be removed, in the majority of cases; although, perhaps, a few years' extension of the term of life may be gained by the operation. The formation of the poison therefore is concomitant not with the adhesive, but the ulcerative inflammation; and it is fair I think to infer that the matter of the poison is generated not by the action which forms the tubercle, but by the series of actions instituted to destroy and remove it. But it is probable that the vessels which deposited the tubercle are the parents of those which nourish it, and from which, in the ulcerative stage of inflammation, the morbid secretion is derived." 225.

We before observed that, in medical reasoning, theories, however brilliant, and subtleties, however fine, must be tried, after all, by the common experience of common men. Is it, then, the fact that persons *generally* and *finally* recover, in whom the disease is freely removed in its early stage? This is a question not to be decided by rules of logic nor laws of ratiocination, but by experience, and by nothing else. Mr. Travers, of course, believes in the correctness of his statement, and certainly he must have seen a great deal of the disease. Others, however, of equal experience with himself, have been constrained to draw a more gloomy picture of the powers of the operation whenever had recourse to. Mr. Travers imagines that the system is not contaminated till the scirrhous tumour begins to ulcerate in its centre, or that the matter of the poison is *generated*, not by the action which forms the tubercle, but by the series of actions instituted to destroy and remove it. This is ingenious, but then we must step over a difficulty to get at it—*what*

*formed the original scirrhous tumour?* The "constitutional contamination" from ulcerating scirrhus, is nothing more than the deposition of that scirrhous tubercle in other parts, *in its primitive, and, according to Mr. Travers, its non-malignant form.* See in what confusion we become entangled by acceding to Mr. Travers' hypothesis! The tubercle is not malignant till it ulcerates—the constitution is then affected, and forms, what? malignant, or, in other words, ulcerating tubercles? No—tubercles in their adhesive and healthy stage. Thus, the effect and the test of *malignancy* is the greater or less universality of depositions *not* malignant.

Those who deny the malignant character of scirrhus from first to last, must necessarily get into such awkward scrapes as the above. It is safest and wisest to allow that scirrhus is essentially a malignant disease, keeping our eyes open, at the same time, to the fact of its occasional, though far from frequent, cure by operation. Before closing this part of his paper, Mr. Travers alludes again to the "medullary cancer," and hints at a connexion between it and scrofula. We must now, however, pass to the second portion of the Essay, which treats of the various situations in which these malignant diseases may appear. This promises to be the most valuable, because the most practical division of the subject, and we hail a general and complete history of the malignant tumours that attack the various compartments of the human frame, as an inestimable boon to the profession. Mr. Travers proposes to treat of these diseases in the following order:—1st. Of the malignant diseases of the face and head. 2d. Of the malignant diseases of the external conglomerate glands, viz. the salivary, mammary, and testicle. 3d. Of the malignant diseases of the organs of generation in both sexes. 4th. Of the malignant diseases of the trunk, including the viscera; and the extremities. Under each division, he will shortly notice the diseases liable or likely to be confounded with these, their more obvious modifications by varieties of texture, and, lastly, observations on their medical and surgical management, will form the conclusion of the memoir. The plan is a noble one, but too extensive, we fear, to be carried into effect by any one man in the way which it deserves. The execution would embrace one third of the practice of surgery, and no inconsiderable share of that of physick. We wish our author well in his arduous task, of cleansing this Augean stable of the filth and rubbish that the vague observations and erroneous notions of more than 20 centuries have amassed and may perpetuate.

### MALIGNANT DISEASES OF THE HEAD AND FACE.

### 1. *Cancer of the Face.*

"The malignant ulcer of the integument of the face commonly begins in a small warty tubercle, hard, irritable rather than painful, sometimes discoloured, so as to look like a dirt-spot. It is usually seated upon the side of the face, upon or between the zygoma and base of the lower jaw. It is also met with, though rarely, on the forehead, lacrymal, and infra-orbital fossa, and sometimes the chin. In the first stage it is slow—noticed even for years before it arouses the alarms of the patient. This is especially the case when obscured by the whisker, or situated in parts not obnoxious to the razor. When fretted by frequent handling or wounded, or irritated by caustic and stimulant applications, it inflames superficially and becomes exulcerated, discharging a thin matter and scabbing by turns. It next acquires a broader base of induration, has a livid circumference, and an even and glossy surface of an unhealthy brightness. There is an occasional sense of heat and soreness, but not amounting to pain. The health continues unaltered. The third stage into which the disease shifts, is that of extensive ulceration both in breadth and depth; the ulcer having an irregular margin and surface, and a profuse suppurative discharge of a peculiar odour. Exuberant fungous granulations are intersected by deep interstices or hollows, in which are lodged ash-coloured sloughs of the exposed fascia, muscles, vessels or nerves of the part. The countenance becomes disfigured by the encroachment of the ulcer upon contiguous parts and by partial paralysis. The pain is now frequent, if not constant, burning and shooting. The complexion, strength, and flesh undergo a gradual, but sensible change—the mind becomes irritable and anxious—appetite and natural sleep fail, the pulse is rapid and small, and spontaneous bleedings take place at intervals. Ultimately death ensues from exhaustion owing to interrupted deglutition and continual irritation; and for the most part it is accelerated by repeated loss of blood." 230.

The patients whom Mr. T. has seen have generally been florid and robust, seldom affected in their general health till the advanced stage of ulceration, sanguine of recovery, and, therefore an easy prey to the bold bad quack, either in the profession or out of it. Mr. T. would say it was more incidental to the inhabitants of the country than of the town, as well as to the ages of from fifty-five to seventy, than any other period. The sore is a specimen of the most purely local cancer, and long after ulceration remarkably circumscribed. Caustic is strongly reprobated by our author, who observes that the proper remedy is free excision, both in breadth and depth, of the indurated wart or tubercle in the skin of the face, occurring at the stated and critical period of life. The absorbent glands are seldom so affected before ulceration as to contra-indicate the operation. In the stage of ulceration, when Mr. Travers thinks that its characters are too well marked to admit of a mistake as to its nature, a cure is hopeless, irritating applications destructive, and soothing ones only admissible. Such are, the watery infusion of opium, infusion of hemlock, &c. under a simple emollient or poultice, and the properly diluted ointments of the oxydes of bismuth and of zinc. In the stage of superficial ulceration, transient and fallacious improvements not unfrequently occur. The disease, on the whole, is not very common.

2. *Medullary Tumour of the Face and Angle of the Jaw.*

This is sometimes seated in the cellular membrane, more commonly in the lymphatic glands. Mr. Travers has seen it occupying the situation of the zygomatic fossa, and also over the parotid gland, covering this and a portion of the buccinator muscle. Such tumours are moveable at first—of slow growth—little painful—increasing, from the size of a small bird's to a goose's egg, when the skin is universally adherent, erubescent, and polished. Their softness and appearance of pointing induces the inexperienced surgeon to puncture them, when blood, not pus, escapes. Mr. Travers has seen them situated on the hairy scalp, when "the subjacent bone is affected by the disease, or ulcerated by pressure before they reach any considerable size." Our own observation would lead us to conclude that, when the medullary tumour appears on the scalp, it *originates* in the cranial diploë. We could mention some curious cases, did our limits allow of the digression from analysis. The lymphatic gland over the parotid, and the glands at the angle of the jaw, are not unfrequently the seat of the medullary tumour, which, on section, exhibits a compound character, a mixture of the degeneration and the normal glandular texture. Mr. Travers mentions three cases of the disease in the several situations above-mentioned, in all of which the operation failed.

*3. Cancer of the Eyelids and Contents of the Orbit.*

"It begins in the form of a hard fretful pimply ulcer upon either palpebra, or one of the borders or angles of the tarsi. It is discoloured by inflammation and sometimes itches, discharges a thin matter, and scabs repeatedly. When it draws surgical attention it is an irregular sore, notching or puckering the border of the affected lid by removal of its substance, and creeping around the orbit. Its progress is slow, but after some time the conjunctiva of the palpebra becomes elevated, thick, and rigid. The ulcer at length environs the orbit and eyeball, and a luxuriant fungus overshoots, and together with the hanging remnants of the lids, buries the eye—so that, although the globe remains, it becomes difficult to be seen. The pain is itching and burning. The ultimate stage of the disease presents a horrible appearance. For a long time the globe remains (I have seen the cornea and humours clear) suspended, as it were geometrically, in the centre of the ruin—the malar and temporal bones are denuded, but an immense fungous mass encircles the orbit, and in part springing from it, is everted over the supercilium, nose, temple, and cheek. The globe at last perishes, but seems rather to yield to its complete insulation than to the destroying process which it has so long withstood." 235.

Two instances of extirpation are glanced at, in both of which the disease returned. Mr. Travers removed the lacrymal gland exclusively affected with scirrhus, and prior to internal ulceration. The patient continued free from disease for some years, but has since been lost sight of.

*4. Medullary Tumour of the Eye-ball and Contents of the Orbit.*

This disease has been so fully described by ophthalmic writers, that our author does not enter into particulars respecting it. He remarks, however, that the peculiar metallo-lustrous, or tapetum-like appearance of the fundus of the eye is *not* diagnostic of the disease, as he has seen several cases in which it has continued stationary, with the eyeball dwindled, a fair presumption against malignancy. In these cases long alterative or salivating courses of mercury had been employed. Mr. T. has also lately seen a case which convinced him that adhesive inflammation of the choroid coat, terminating in a deposit of lymph, which undergoes a vascular organization between that membrane and the retina, "presents an appearance so exactly resembling that of the incipient medullary cancer, as to put it beyond doubt that the real nature of the appearance, if not the occasion, is one and identical in both cases." The case was that of a young lady, in which it followed a wound with a pair of fine scissors, which had passed obliquely between the margin of the iris and the ciliary body.

"The best diagnosis is founded on the increase of volume of the eyeball or the contrary, prior to the giving way of the tunics. Even this, and loss of shape, and discoloured tumours of the sclerotic are, however, insufficient, as all may be produced by disorganizing choroiditis; but the progressive advance of the tumour to the cornea and the shrinking and sloughing of the latter membrane, which happens prior to the protrusion of the fungus, is decisive of all doubts. The hydrophthalmic enlargement, or the direct collapse by interstitial absorption of the contents of the eye-ball, are sure indications that the disease is not malignant." 237.

Medullary cancer is frequently seated in the fatty membrane at the back of the globe.

"An extraordinary globular tumour is formed around the ball, of which the perished cornea forms the centre. It projects, stretching and so separating the lids, that they girt tightly the base of the enormous swelling. I have seen several children the subjects of this affection. The growth is sometimes confined to the upper or frontal aspect of the orbit. The upper lid is then prolonged and stretched over the globe so tightly that it is difficult, if practicable, to obtain a view of the latter. The medullary matter is of a granular or ricy consistence, and pervades and destroys the muscles, periosteum, and finally the bony vault of the orbit. I have seen its extirpation boldly performed; but its re-appearance has been almost immediate, and its progress quick to destruction." 238.

Mr. Travers never knew an instance in which medullary cancer of the eye failed to return after the operation. When firm adhesion exists between the lining membrane of the orbit and tubercles of the globe or the nerve, even to the foramen opticum, the operation is very difficult, and unsatisfactory in its result. Deep-seated disorganizing inflammation is apt to be mistaken for this disease, but the transverse section of the globe is an efficient remedy and a proper test, which should always be practised if a doubt of the nature of the case exist. In the malignant disease, a small discharge of blood and black pigment, or coagula stained with it, ensues, without collapse of the globe; in the other, a discoloured fluid escapes, the globe collapses, and the cure is complete.

5. *Cancer of the Lower Lip.*

"The commencement of this common and well known disease is in the intercellular tissue of the mucous membrane and skin. The enlargement and induration render it conspicuous before the villous surface of the lip cracks transversely, and oozes a thin fluid, then exulcerates and scabs by turns, and ultimately ulcerates deeper and fungates. The patient is generally a healthy male of advanced years, and accustomed to smoking. Pus sometimes escapes on a section of the fungus, but the stool or base of the tumour is hard, granular, or seedy; the skin and mucous membrane and the labial glands, now prominent and warty, are closely compacted together and indurated. As the ulceration proceeds, the induration extends, and the salivary glands beneath, and the lymphatic glands, at one or both angles of the jaw, become sympathetically enlarged and tender.

Healing the sore by escharotics, &c. is tried in vain, and the only rational treatment is to remove the disease effectually and freely in its early stage. This is usually done by the hare-lip operation, but Mr. Travers prefers its excision by a full crescent-shaped section of the substance of the lip, the commissure of the mouth being left, if possible, and no suture required. The contraction and cicatrization take place remarkably well under a double-headed bandage, passing over the vertex and occiput, so as to keep a little moistened lint or simple ointment on the cut surface; whilst the disuse of suture and ligature obviates a very irritating and objectionable portion of the treatment.

### 6. *Cancer of the Alveolar Membrane of the lower Jaw.*

This is a rare, but well-marked form of malignant disease, not described in books, as far as Mr. Travers recollects. Mr. Travers' attention was first drawn to it by Mr. Cline, in a case where he met that able surgeon in consultation, and since that period, he has seen it thrice. All the patients were aged persons, and they die of exhaustion from deficient nourishment, pain and repeated hæmorrhages. The following is the graphic description of the malady.

"It commences at the point of reflection of the membrane of the gum on the alveolus, or on the inner side of the gum at the root of the teeth, where the sore mouth, from mercury, is commonly first perceived. Small granular eminences or tubercles are formed, by which the membrane of the gum is raised and thickened into a small lump. The disease begins, in my experience, about the root of the last incisor or bicuspid, and thence gradually enlarges backwards to the middle molar teeth. Ulceration then ensues, the edges of the ulcer fungating and bleeding frequently,—it is slowly, but progressively phagedenic, destroying the soft parts, and ultimately, by ulcerative absorption, the substance of the maxilla, so as even to divide the bone. The adhesive inflammation of the contiguous membrane and soft parts during this process, and the tumefaction of the submaxillary gland give a peculiar elongation, breadth, and bulging of the jaw on that side, which is a characteristic feature of the disease. The imperfect opening and cleansing of the mouth, the difficulty of taking nourishment, and the horrible fetor of the discharge,—the constant gnawing pain with shoots darting upwards to the temple, render this a disease of great suffering. It admits only of palliation by the frequent use of antiseptic and detergent gargles and lotions, as of lime-water, camphor, myrrh, borax, honey, &c. Oxyphosphat of iron, and compositions of verdigris and caustic, are of no avail. Sarsaparilla dissolved in milk, boiled bread and milk, animal jellies, and soft nutritive mucilages are best adapted for sustenance and medicine." 243.

### 7. *Medullary Tumour of the Mouth and Fauces.*

Mr. Travers has seen several instances of fungoid tumour situated within the cavity of the mouth—on the upper maxilla; on the lower maxilla; and more than one instance growing within and around the incisores teeth, and covering the symphysis of the lower jaw. Extirpation is attended with very free hæmorrhage, and invariably leads to a rapid re-production of the tumour, which proves fatal by impeding, or rather obstructing nutrition. It differs from the scirrhous affection described in the last section, as being a fungous production, not an eroding ulcerative disease.

### 8. *Cancer of the Tongue.*

"This is not a smooth and firm rounded tubercle, such as is often met with in this organ, but an irregular rugged knob in its first stage, generally situated in the anterior third, and midway between the raphé and one edge. It sometimes, but seldom, extends across the middle line, although it often extends alongside of it. The hardness is unyielding, inelastic, and the mucous surface puckered and rigid. It also gives to the finger and thumb of the surgeon the sensation of solidity, or of its penetrating the entire muscular substance, being perceived equally on either surface. Sharp shoots of pain are felt through the side of the affected organ, towards the angle of the jaw and ear. The disease tends to run backward towards the base or posterior edge. It sometimes acquires great bulk before ulceration takes place, so as to project the tongue from the mouth. In this state a female patient of mine was seen some time ago in St. Thomas's Hospital, in whom the permanent projection of the diseased organ beyond the widely distended lips was from three to four inches. Life was sustained for a time by nutritive injections. The ulceration often extends from the edge of the tongue to the membrane of the mouth and gums, when the elevated and distended membrane at length gives way, and ulceration is rapid. The surface of the ulcer is very uneven, clean and bright granulations appearing in parts, and in others deep and sloughy hollows. The darting pain is very acute, but only occasional. There is a dull aching always present, and as constant a spitting as in deep salivation. The irritation is such as soon impairs the powers of life. It happens to strong and hitherto healthy persons, for the most part males from the age of forty onwards. There is generally an evening paroxysm of pain, and the nights are much disturbed by the secretion accumulating in the throat, which excites cough. Often the patient is roused by a painful compression of the tongue falling between the jaws. The leaden hue of the countenance, the loss of flesh, and difficulty of taking food, although symptoms of the advanced stage of the disease, are observed long before the appetite or muscular powers fail in proportion. Frequent moisture with mild fluids, as tepid milk and water, or confectioner's whey, is grateful to the patient. Speech is much affected and painful.

"Towards the fatal termination of the disease occasional profuse hemorrhages take place at shortening intervals, and alarm and weaken the patient, who ultimately dies tabid and exhausted, generally with symptoms of more extensive disease of the mucous membrane in other parts." 246.

The period at which the sublingual and contiguous lymphatic glands become affected, as well as the extent of their implication, vary. Local irritation from the pressure of a tooth is an ordinary exciting cause, in the same manner as the pipe in cancer of the lower lip. An accurate diagnosis between this and the other affections of the tongue is an object of paramount importance, from the early and effectual removal by the knife or ligature, when possible, being the only chance for the patient in the genuine scirrhus of the tongue. Mr. Travers, however, has only seen one instance of the operation not being followed by a recurrence of the disease within a twelvemonth, a melancholy fact indeed. The needle, when the ligature is employed, (and excision is hardly safe, when practicable) must be passed beyond the centre of the disease through the sound parts, armed with a double thread. The cautery, caustic, and all stimulant applications, even borax, are mischievous; the carbonate of iron and alkaline carbonates are of no benefit, but the black wash, on the whole, is the best application. Mercury, iodine, arsenic, &c. have no permanent influence over the disease.

### 9. *Cancer of the Antrum.*

"This most disfiguring and destructive disease begins upon the lining membrane, and first shews itself in a bulging of the cheek under and upon the malar bone. The tumour is elevated, circumscribed, and hard, and the integument has a blush of colour. The pain is inconsiderable when the patient is alarmed by the appearance and increase of the swelling. The nostril soon becomes closed on the same side, and the teeth loose; they fall out, or are extracted, and a copious oozing of purulent ichor takes place into the mouth. The introduction of the probe by the nostril or palate is followed by free bleeding. If the alveolus is trephined, a fungus shoots up, fills the opening, and covers the gum. Next, the palate becomes depressed, so that the arch on that side is lost, and either the eye-lids are closed, or the eye protruded; and completely amaurotic in either case. In the mean time the external swelling gains size, is quite immoveable, and the skin acquires a livid hue. Small veins are seen rampant upon it in great numbers, forming a net-work. There are commonly one or more depressions where the bone is absorbed. These break and discharge pus. The patient suffers a good deal of burning and darting pain. The ulceration extends until the mouth communicates directly with the surface, and fluids escape from the wide opening in the cheek, which is surrounded by a raised thick everted border of granulation; or the openings are less direct and fistulous, in which case the tumour acquires an enormous bulk, and the roof of the mouth is upon a level with the incisor teeth, and compresses the tongue. I have seen the disease extend its ravages in the direction of the nares, and destroy the nostril on the same side, instead of the cheek. It is needless to add, that the distress of the patient from the noisome discharge, which is very abundant, the increasing difficulty of taking sustenance from inability to masticate and fear of choking, the consequent emaciation, and loss of rest from the presence of constant febrile irritation, are soon destructive. Hemorrhages and colliquative sweatings generally hasten the desired event." 250.

Mr. Travers has seen this disease arise in an elderly lady who had large, old, ulcers on both legs, which she avoided healing up entirely, and indeed could not. On examining these tumours, they are found to consist of a chaotic mass of coagula of lymph and blood, holding spiculæ of bone. Patients frequently, but no doubt fallaciously, refer the origin of the disease to the extraction of a molar tooth.

### 10. *Cancerous Fungus of the Nares and Antrum.*

A growth essentially malignant, but happily rare, to which the common lining membrane is subject. It is a brittle fungus, extremely vascular, growing from the whole surface of the cell, most commonly in the antrum, distending the parietes enormously by its rapid growth, and reproduced again and again with great celerity after masses of it have been cut or torn away, the separation being generally followed by excessive or even dangerous hæmorrhage. It is denominated, improperly in Mr. T.'s opinion, malignant polypus, and is sometimes confounded with the fleshy polypus.

### 11. *Cancer of the Fauces and Pharynx.*

Scirrhous tonsil, like many other things in surgery, is much talked of but little seen. But the broad papillæ at the root of the tongue, the tonsils, and the mucous follicles of the glottis and pharynx, are each occasionally the proper seats of the disease, beginning in tumour and induration, and terminating in fungus.

"Scirrhous strictures followed by ulceration and cancerous fungus are met with in the pharynx and top of the œsophagus in elderly persons, chiefly females in my experience. They are productive of constant nausea, dry burning sensations in the throat and stomach, difficult breathing, frequent spasms and alarms of suffocation, and excessively impeded deglutition; upon the gentlest introduction of the finger or bougie, hemorrhage follows, which afterwards becomes spontaneous. The patient has a faded sallow countenance, a disturbed circulation, and is emaciated to a skeleton." 252.

### 12. *Cancer of the External Ear.*

The ear is rarely primarily affected, but Mr. Travers has once seen the upper third of the external ear the exclusive seat of an indurated sore, having every character of cancer. The diseased piece was amputated, the wound healed, and the patient, Mr. T. believes, remains sound.

### 13. *Medullary Tumour of the Internal Ear.*

Mr. Travers has seen but one example of this disease. The tumour extended from the temporal fossa to the angle of the lower jaw, and internally to the posterior nares and fauces. The mastoid cells apparently escaped. The jaw became locked; a bleeding fungus filled the meatus; the head, face, and muscles of deglutition were paralysed; coma succeeded; the patient was nourished with great difficulty, and inanition accelerated his death. No post-mortem inspection was permitted.

### DISEASES OF THE HEAD AND FACE, SOMETIMES MISTAKEN FOR CANCER.

The three first affections described under this head are, the crustaceous herpes, lupus, and a peculiar affection of the integuments of the face, resembling elephantiasis. These affections being described in the works on cutaneous diseases, we shall pass over here, and stop at,—

### 4. *Ulcers of the Mucous Membrane of the Mouth and Tongue, Fauces, Nares, &c.*

"From these there can scarcely arise any difficulty in diagnosis. They are aphthous or tuberculous, scrofulous or venereal, or of the herpetic character which I have described as affecting the skin of the nose and upper lip especially, and there forming a scab or crust. I have seen the pits or fissures of the tongue following and even alternating with this herpes of the nose. A lunar caustic lotion, or the verdigris liniment, or the caustic itself in pencil, are the best applications, and arsenic, steel, and sarsaparilla the best medicines. Mercury is decidedly injurious. They are as little venereal as cancerous, but they frequently follow mercury, and are indeed a symptom, among many others, of mercurial scrofula.

"The tongue and lips are not unfrequently the seat of venereal ulcers, as well as the nose and palate. These are secondary sores, with few exceptions. Sometimes however, the tongue exhibits a genuine chancre, although it is not easy to explain the occurrence even among the most abandoned. The appearance presented by the large and deep fissures of the secondary ulcers, with their high edges falling asunder, and the irritable and painful state of the surrounding parts, the sensible increase of destruction from day to day, and the fetid discharge mixing with the saliva, lead some to suppose them cancerous; whilst others—from their invariable aggravation by mercury in any but the mildest form, and unaided by generous diet and sarsaparilla—regard them as cachectic, and the result of mercurial action in a scrofulous habit. The employment of mercury requires greater delicacy both in the time and mode of exhibition in sores, whether venereal or cachectic, affecting the lips, tongue, and palate than similar sores in any other region of the body. In many it is contra-indicated altogether, while to others it is essential, even when the history renders their venereal origin disputable. But to pursue these observations would lead me too far from my subject." 258.

### 5. *Globular Tumour of the Tongue.*

This is like a marble in size and touch, situated deeply in the substance of the organ, very uniform and unyielding in its surface. It is occasionally mistaken for scirrhus, but invariably disappears under the use of medicines calculated to improve the tone and secretions of the stomach, as the alkalies, with bark and steel. From its complete absorption Mr. Travers supposes it to be a cyst containing an albuminous fluid, but he has never wounded it.

### 6. *Vesicular and Fleshy Polypi.*

The vesicular, hydatid, or common polypus of the nares is too well known to require description : it is not liable to the suspicion of malignancy. The fleshy is flat, oval, or oblong, like a horse-bean, solid, opaque, and generally has a broader attachment than the former. It is most common in the posterior nares, where it is productive of much inconvenience to the patient. Many may exist together, and being in firm masses they do not break readily.

"Polypi are frequently formed in the meatus auditorius. When springing from or contiguous to the membrana tympani, so as to prevent its vibrations, they occasion complete deafness. There is a vesicular polypus auris, which takes the shape and length of the meatus, and with ordinary caution is extracted entire, like that of the nares. The fleshy is granulated, very vascular, and brittle, lies upon and conceals the drum, and does not grow to any great size, but ulcerates and gleets profusely and offensively. This requires cautious treatment. I never saw any possessing a character of malignity.

"Polypi of both species form in all the cells and chambers of the bones of the face and head. The lachrymal fossa and inner angle of the orbit, above and below the tendon of the orbicularis palpebrarum, is the place next in frequency to the nostril, at which they give external demonstration of their existence. Next to this, the antrum ; and, indeed, the disease in the antrum generally presents these appearances in the nostril and at the orifice of the lachrymal canal, before that chamber is bulged externally.

"Polypi occasion the same deformity by their increase in inaccessible situations, as was described in speaking of the malignant tumour of these parts. In the antrum, they protrude the cheek, lift the floor of the orbit, obliterate the nostril, and depress the vault of the palate. In the orbit they protrude the eyeball, and projecting on either side, fix and compress it in the opposite direction. In the æthmoid, sphenoid, and frontal sinuses they loosen and even detach the bones from their connexion, and occasion a hideous deformity, with paralysis and coma, the consequences of serous effusion in the ventricles of the brain." 260.

Mr. Travers considers both vesicular and fleshy polypus as originally quite free from malignancy, but occurring in the deeper and inaccessible parts they must prove ultimately destructive from their productive and reproductive tendency, progressively extending from cell to cell, and occasioning by their growth the formidable consequences already narrated.

"The cancerous fungus which I have described as growing from an extended surface, not a point of the membrane of the nares or antrum, and which, by this circumstance, is marked as a truly malignant disease, I have heard surgeons denominate 'fleshy polypus ;' with which disease it has nothing in common but its effect to distend and burst asunder the parietes within which it is contained. The character of the deep ulcerated openings which form in cases of polypous growth, and the heterogeneous mass left after the process is terminated by death, the sufferings of the patient, the noisome fetor of the discharge, and the frequent and free hemorrhages preceding dissolution, which are incidental, not characteristic, features of analogy, have led some observers to conclude that the disease, although simple in its commencement, has taken on or induced a cancerous action. But I doubt if cancer is ever a consecutive disease; although I know many instances of its being chronic, and of cancer being set up at a particular period of life in persons who were the subjects of other disease, and of local injury, seeming to afford the occasional cause of its appearance." 262.

This brings the present paper and our analytic labours to a close. The remaining divisions of the subject will be discussed by Mr. Travers at a future opportunity, and when that opportunity is seized we shall take especial care to bring the learned and talented author again before our readers. Our opinion of the merit and substantial value of the memoir from which we are now to part, is stamped in the copious analysis we have given of it; for we may say of our respect for the productions of writers, as the GIAOUR says of his passion on his shriving day:—

"But these are words that all can use,
I proved it more in deed than word."

As it is customary, however, in good company, to salute on taking leave, we beg to make our *congé* to Mr. Travers, and return him many thanks for the instruction we have received in perusing his account of the various malignant diseases of the face and head. We are convinced that if he prosecutes the subject, and describes, as he proposes, the same diseases in the other portions of the body, he will do an essential service to the profession, and no doubt obtain, as he will deserve, its gratitude.

